# Study of Rotational Motions Caused by Multiple Mining Blasts Recorded by Different Types of Rotational Seismometers

**DOI:** 10.3390/s21124120

**Published:** 2021-06-15

**Authors:** Krzysztof P. Teisseyre, Michał Dudek, Leszek R. Jaroszewicz, Anna T. Kurzych, Leopold Stempowski

**Affiliations:** 1 Institute of Geophysics, Polish Academy of Sciences, Księcia Janusza 64, 01-452 Warsaw, Poland; stempowski@igf.edu.pl; 2 Institute of Applied Physics, Military University of Technology, gen. Sylwestra Kaliskiego 2, 00-908 Warsaw, Poland; leszek.jaroszewicz@wat.edu.pl (L.R.J.); anna.kurzych@wat.edu.pl (A.T.K.)

**Keywords:** rotational seismometers, optical fiber sensors, seismology, rotation rate, symmetric strain

## Abstract

Digging two vertical shafts with the multiple blasts technique gave the opportunity to measure the induced angular motions in a horizontal plane with well-defined positions of sources. Three kinds of rotation rate sensors, sharing an underground location, were used. Two of them—a Fiber-Optic System for Rotational Events & phenomena Monitoring (FOSREM) and a prototypical seismometer housing the liquid-filled torus—sensed the rotation, while a microarray of two double-pendulum seismometers sensed both the rotation and symmetric strain. The FOSREM was sampled at 656.168 Hz, while all the others were only sampled at 100 Hz. There were considerable differences within the results gathered from the mining blasts, which should be attributed to two causes. The first one is the difference in principles of the operation and sampling rates of the devices used, while the other is the complex and spatially variable character of the studied wave fields. Additionally, we established that the liquid-filled sensor, due to its relatively low sensitivity, proved to be viable only during a registration of strong ground motions. Overall, a comparative study of three different rotational seismometers was performed during mining-induced strong ground motions with well-localized sources.

## 1. Introduction

Mining works give an opportunity for engineers and geophysicists to acquire valuable knowledge on the substrate response and the ever-changing possibilities of recording and measuring devices used to monitor the process. The physical aspects of an explosion include the creation of elastic waves, and this kind of physical interaction is the area of our investigation in this paper. Elastic waves, also named seismic waves, in cases where the ground, body of water or, in general, part of the Earth or other celestial body is affected, divide, in turn, into body waves and boundary waves (also named surface waves for the atmosphere–ground boundary). It should be noted that so-called detonation is an explosion that also generates shock waves that propagate extremely fast [1,2], and these were not studied in this case.

The idea of applying multiple blasting is to arrange the explosions in a spatiotemporal pattern where the energy is directed inside the chosen volume in as big of a proportion as possible to crush the material effectively and economically. In the works studied here, in most cases, a bigger number of charges were located peripherally, each of which were usually smaller than the packages from the central group. Such patterns, which enable the restriction of the damaged area, are widely used.

The methods of multiple blasting have been developed by theorists and practitioners as well [3], while the goal is to restrict the damaged area and to minimize the unwanted vibrations outside. For example, in Reference [4], which concerns various mining works during a tunnel excavation, the explosives arrangement was sketched, resembling a wide horseshoe or arc, again with the peripheral charges ignited last. Additionally, in Reference [5], the pattern of firing the explosives in the course of underground mining was discussed. The study included the blast-induced vibrations above in the village where these were recorded. Here, the dominant frequencies were found in the range from 30.1 Hz to 246 Hz at the surface distances reaching 300 m. The maximal ground velocity found in these measurements was in the range of 2.34–14.6 mm/s.

Three components of the peak ground velocities in the quarry blast-induced seismic vibrations were reported to attenuate dramatically in the close distance to the source, up to 80 m [6]. Additionally, at these very close distances, the damaging effect of seismic waves is maximal and dependent on the kind of exploited rock. For greater distances, let us quote the authors: “Propagation of the [particle] velocity is not dependent on the type of deposit and the environment in which the seismic wave propagates”. According to the same authors, it is well-known that the same amount of fired explosive may cause different maximum ground particle velocities; the factor of variability exceeds 3.

In the mining works, blasts are used to crush the rock, while, in engineering and technics, there are many uses for them also in demolition and rescue actions [7,8,9]. Elastic waves, both created with blasts and generated by ubiquitous processes of stress accumulation and their violent relaxation, taking place in mines are of great importance as a fount of information on seismic event sources and on hidden structures as well. Such studies enable the prediction, e.g., of the peak ground velocities and translational and rotational as well, based on the measurements with six degrees of freedom [10]. Vibrations at the surface and underground are also studied as concerns the aspects of houses’ and other facilities’ safety and the welfare of people and animals [11].

In recent times, explosions have started to be replaced in mining works by new methods, less dangerous and making less trouble for the environment: pouring flammable medium into the rocks with the use of numerous drilling and then burning it, then the expanding gases crush the rock [12]. Additionally, the methods to control the blasts and to measure their effects are still advancing, as are the methods for the gathered data analysis.

Studies on both vibrations and permanent deformations embrace, more and more often, monitoring, analyzing and modeling rotational movements, although these activities are still in the initial developmental stage. As it was stressed by Zembaty et al. [13], the areas of induced seismic effects are potential test fields of earthquake-proof and, also, rotation-proof engineering. These authors analyzed 51 records obtained in the period of two months from the surface station in the deep mining area of Ziemowit Mine, located in the Upper Silesian Coal Basin in Poland. They discussed in detail the three strongest shocks; additionally, the rotational and translational velocities and accelerations were compared. Problems of the proper classification of the rotational motion strengths were also raised.

Rotations are usually found at the beginning of records—see the short resume of this difficult issue by Fichtner and Igel [14]. These motions—together with symmetric strain motions, which are also angular, not rectilinear—were very important components in the near field [15], where Fuławka et al. [10] generally found 1.5–2-times higher frequencies in the recorded rotational motions than in the translational ones (taking into account only the high-energy events), though there were exceptions.

The rotational components and effects are still being studied, mainly in the horizontal directions. Nonetheless, some seismic engineering investigations have dealt with the vertical aspect of these phenomena and with the three axes of rotations—see, e.g., Reference [16], where the eigenmodes of the oscillations measured in high-rise Moscovian (mainly) buildings were studied, and these oscillations proved to comprise the rotational parts. Additionally, the seasonal variations and long-time creeping of these eigenmodes were noticed in that work; moreover, the influence of our planet’s own oscillations on the results was detected.

It should be underlined that the rotations present in the seismic wave field are still interesting per se, not well-known and not sufficiently popular as a theme for studies. This is probably for two reasons: firstly, the technical difficulties in the research, and secondly, these motions and deformations do not match, at least according to many scientists, the dominant model of continuous solid bodies (and of the Earth itself in its solid part). This model is named the Elastic Continuum Theory (or the elasticity theory) and belongs to the basics of the solid-state physics, geophysics and technical sciences [17]. The elasticity theory embraces only two categories of mechanical deformations and motions in continuous solid materials: rectilinear (the volumetric included) and the symmetric angular ones. The latter are also named shear or the shearing motions and deformations. For rotary motions and deformations, there is no place provided in this theory; only a role of chaotic, local inhomogeneities and disturbances is ascribed to them. Nevertheless, as was explained by Cochard et al. [18], rotations are present in the far field of seismic body waves, as the aspect of the S-wave component of the field. This was later extended for near-field conditions, where the spherical wave front is taken as a sufficient approximation [19,20]. These findings allow to quickly assess the phase velocity of the wave. Generally, the elasticity theory-based approach allowed us to develop methods improving the seismic surveys and the monitoring practice. If rotary motions and deformations are taken into account, together with the rectilinear ones, then scientists and engineers obtain a wealth of additional information and, e.g., this may serve for ‘S-wave tomography‘, which is possible even with use of only one station [14,21].

Beside the progress in using the measurements of rotations (and the symmetric strains) in geophysical practices, many scientists have developed theories of a such solid continuum that has properties transcending the elasticity theory, hence allowing for rotations inside a solid material. These postulated rotary motions should also be express in large-scale rotary motions and deformations. These theories, known generally as micropolar elasticity or Cosserat continuum theories [22], received experimental confirmation in the laboratory [23,24,25], though, as our knowledge permits us to state, not in field studies. Mathematical modeling of the micropolar continuum, which is the medium consisting of interacting particles (such as grains or sub-grains) or of functional structures, often called shells, is also progressing. Postulated mutual interactions include friction, sliding and passing deformations and rotations [26,27,28]. To date, other theories still belong to the asymmetric continuum theory [29], which is based on the concept that, in a rock mass excited, e.g., by an earthquake, two kinds of motions interact: one is a shearing motion and the other a rotational one, and the interplay of these two motions enables their joint propagation.

Probably the broadest range of different ideas among the seismic rotations and of their various studies collected in a single volume is found in two monographic editions [30,31] with a good introduction in Reference [32]. Additionally, the important geophysical appliances of the Cosserat continuum belong to the studies and a new theory presented in Reference [33], which shined new light on the reactions and relations of joining faults in the Earth’s crust. According to this book, the spatial and temporal relations in fault behaviors point to the Cosserat continuum properties of the Earth’s crust and to the Cosserat mechanics of faults. This gives hope for a better understanding of earthquake sequences and hopefully brings mankind closer to an earthquake prognosis.

A very useful approach to passive and active seismic studies is the simultaneous application of various monitoring techniques. First, Suryanto et al. [34] showed a conformity of rotations (coming in waves excited by distant earthquakes) measured with two methods: with the array of seismometers and a rotational sensor (Sagnac type, located in the central position). From this time, the practice of including backing or mutually supporting methods started to flourish. The rotations and translational motions in near-fields were studied in the frame of the TAIGER active experiment performed in Taiwan in 2008, and the subsequent data analysis gave a bulk of data [35,36]. Tilt oscillations caused by wind and the method to remove their influence on seismic recording data by using simultaneous measurements with the rotation sensor (which worked as a precise tiltmeter) were also presented [37]. Lately, the experiments in Fürstenfeldbruck, Germany [38,39] allowed us to compare the many methods of rotation measuring in the field and to formulate sound conclusions, among others necessity for the certain uniformization of these techniques.

It should be noticed that, in the case of a multiple explosion, the source of seismic waves is probably as complex as in a natural earthquake, though many times smaller. Therefore, a manmade source may well generate complicated wave patterns with the acting rotations. Scientists still do not know the exact processes acting on the earthquake source and its vicinity, and there are many different earthquakes and explosions.

Against this background, mining works in the area of Książ Castle, where the geophysical observatory is located and where, underground, different seismological devices, including rotational seismometers, are stationed, gave the opportunity to study rotational and symmetric strain excitations with well-defined sources. The already-known sensors were compared here in a new situation, and the results of this field study were hard to predict.

## 2. Area of Study

**Figure 1:**
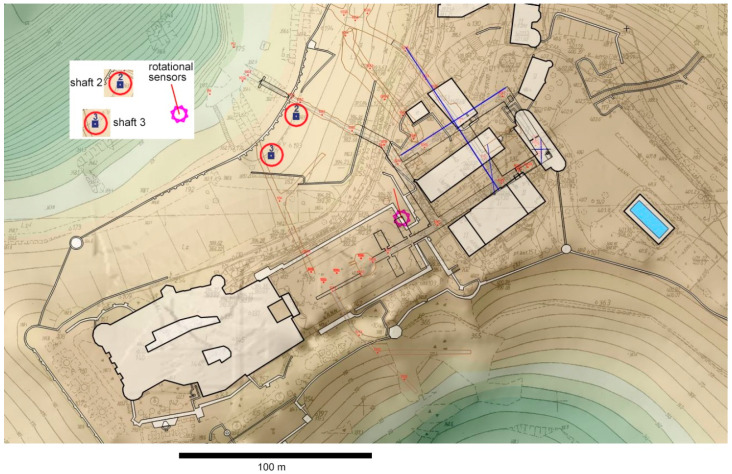
Plan of Książ Castle with its surroundings and identification of the shafts and rotational sensor locations.

The festive castle of Książ in the town of Wałbrzych, Lower Silesia, Poland entices many tourist groups, both from Poland and abroad. The underground at Książ lies in close vicinity to the castle northeast of its main building and consist of a system of corridors and several adjacent chambers, as presented in Figure 1. The height of the underground is of about 5 m, and their floor is almost horizontal.

In the spring and early summer of 2018, in direct proximity to the castle, two vertical shafts, leading to the underground, were dug. These were done using numerous multiple bursts. The aim of this whole venture was to facilitate the growing tourist traffic. It should be noted that this work generated very strong oscillations that encountered the underground at Książ, with the peak velocities of the ground motions greater than usually encountered (mainly of copper and coal mining provenance). In the time of digging, the two entrances under construction were temporarily named “Shaft 2” and “Shaft 3”. Both were vertical shafts about 10 m deep. The distance between their centers was about 25 m, and the horizontal distance to the rotational seismometer location was around 75 m from the center of each shaft—see Figure 1. The final horizontal cross-section of each shaft was a rectangle of 5 m × 3.5 m. The thickness of the ground above the underground varies from about 10 m in the area of the new entrances to more than 40 m in the place where the rotational sensors stay below the border of the castle courtyard.

## 3. The Mining Works

The two new entrances were obtained by using modern mining works, including more than 70 blasts. Almost each of these consisted not of a single explosion but of a quick series of neighboring explosions. Each multiple blast took place at one of the shafts, not at both at once, and it was applied either from below or from the upper surface; there were no mixes. The desired intervals between the firing of individual charges, or their groups, were obtained by using electronic detonators with various time lags. Multiple blasts consisted of 4–30 explosions, usually in groups sharing the same time. The time lapse between “elemental blasts” may be either 25 ms or a multiplicity of this time. The detonators used were adequately named; the smallest lag was labeled “1”, while the greatest was “15” and gave a time delay of 15 × 25 ms = 375 ms. The variance of the time lags applied in these works was considerable, up to 15 delays. The explosive material used was Ergodyn 22 E (Nitroerg S.A., Poland). Individual charges weighed from 30 to 450 g, and the total mass of the material used for one (multiple) blast varied from 30 to 12,750 g. Blasts were applied either vertically from above or from below—in such cases either vertically or at various angle(s).

**Figure 2:**
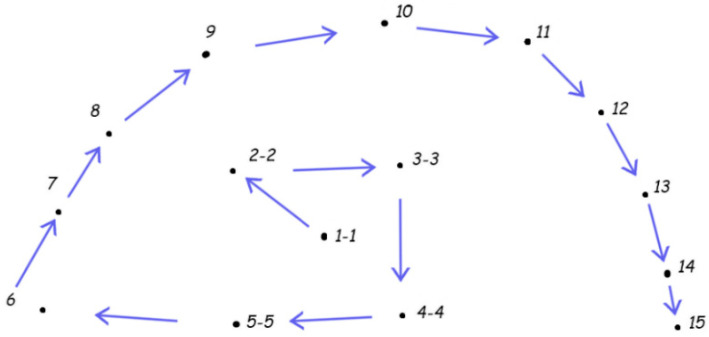
Arrangement and time sequence of the exemplary multiple blast; horizontal cross-section of the location. Sequence of the elemental shots is shown with the arrows. At first, the five pairs of charges were ignited; these were situated at the center.

In the multiple blasts, at first, the centrally placed charges were ignited; then, the peripheral ones. The typical arrangement of the charges is depicted in Figure 2, taken from the documentation of one of the blasts (No. 2, 14 April, 12:03 UTC). The time sequence of the elementary blasts corresponded to the increasing queue; double numbers denoted doubled charges ignited simultaneously. Thus, the latter blasts “encapsulated” those thst were in the beginning (and restricted the crushed area). Since, in this example, there were 15 delays, the total blast time was 350 ms plus the duration of the last elemental blast. The rock mass reaction was, of course, longer. Generally, up to 30th May, for particular multiple blasts, greater and greater masses of explosives were used.

In this article, we mainly explore the recordings of seismic waves excited by sixteen blasts directed vertically from above either in Shafts “2” or “3”. Several blasts chosen from the overwhelming majority of those directed from below were also studied. Here, the most powerful blast was when 12,750 g of Ergodyn were ignited, and the weakest single explosion was of a mere 73 g aimed at crushing a boulder that fell from the roof and lie in the corridor.

## 4. Measuring Devices

For this study, the same rotational sensors were used that were dedicated to the long-time observations of mining shocks in the area and to the technical tests. They were three entirely different one-axis (vertical) rotation-sensing devices. All of them have been previously thoroughly characterized to obtain their fundamental parameters [40]; therefore, below, only the important information about them is presented.

**Figure 3:**
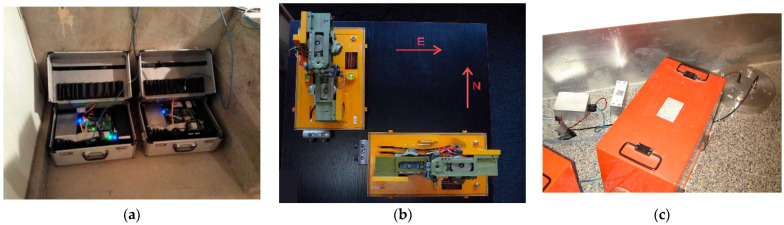
Photos of the rotational seismometers: (**a**) FOSREM, (**b**) TAPSs with open boxes on a table and (**c**) TAPSs (red boxes) and the CZ sensor (silver, to the right) at the location in the Książ underground.

The first of them was the Fiber-Optic System for Rotational Events & phenomena Monitoring (FOSREM), already extensively described [41,42]. The FOSREM is a fiber optics-based rotational seismometer utilizing the technical realization of the interferometer based on the Sagnac effect. This instrument is constructed by applying a minimum open-loop fiber optic gyroscope configuration, where the Sagnac effect produces a phase shift between two counter-propagating light beams proportional to the measured rotation [43,44]. Therefore, it takes advantage of the fact that the rotational phenomena are recorded as sudden changes of the rotation rate, with an amplitude directly calculated from the detected Sagnac phase shift [45]. The main advantage of this approach is its insensitivity to linear motions and a direct measurement of the rotation rate. The sampling rate of FOSREM is equal to 656.168 Hz, and its theoretical sensitivity is at the level of 2 × 10^−8^ rad/s/√Hz. Two such devices were placed on the ground in the chamber near the other seismometers to ensure conditions similar to those for the other seismometers, as shown in Figure 3a, although, due to technical difficulties, only the results from one of them were presented in the current study.

The second kind was the microarray—a set of two twin pendulum seismometers, described already several times [46,47]. It consists of two portable boxes, perpendicularly situated along the east–west (the first one) and north–south axes (the second one), as shown in Figure 3b. We would like to add that, in the mentioned papers, for rotation, the name ‘spin’ was also used, and the symmetric strain was dubbed ‘twist’. Each box contained (see Figure 3b) two mechanisms of the old-time SM-3 seismometers; these shared a common metal rectangular plate that was connected with three vertically adjustable feet to the pedestal. The mechanisms were mounted in horizontal antiparallel positions, prepared to react to the ground motions along the vertical axis. One of these mechanisms was mounted on an elevation in such way that the axes of both pendula motions were parallel. In each instrument, the elevated one was the outer one (in relation to the cross-point of the perpendicular directions at the horizontal surface that supported both devices). The official name of the individual device is the “Rotational seismometer SR-H”, but it is commonly referred to as the TAPS—Twin AntiParallel Seismometer. Shortly speaking, a set of two TAPSs work in the Książ underground, and we had four channel recordings from them: channels 1 and 2 were from the first seismometer; these sensed motions in the north–south direction, while the remaining pair of signals was provided by the second device, which was due to motions in the east–west direction. From these four channels, both the rotation and the symmetric strain were derived in an undirect way, the latter related specifically to the N–S and E–W coordinate system. The functioning of TAPSs, with a possibility of errors, was discussed in detail in Reference [48].

Finally, a disc-shaped (Figure 3c) seismometer with its torus full of silicone oil, RS.LQ–RP/P, further referred to as the CZ sensor, is a sensor developed at the Institute of Geophysics, Czech Academy of Sciences, Prague. Its authors are Dr. Petr Jedlička and Dr. Jan T. Kozák, who jointly constructed a series of prototypical rotational seismometers [49,50]. When the ground rotates, the fluid (silicone oil) inside the torus does not momentarily associate fully with the movement of the rigid construction. This difference in motion generates an electric signal—in the case of this model, by the motion of a paddle-like obstacle immersed in the liquid agent.

All the rotational sensors shared the location in the underground corridor chamber, which had a tortuous connection to both places where the works proceeded. FOSREM stood on the concrete floor, and the others shared the same concrete pedestal, cemented to the rock and topped with a granite plate smooth at the upper surface. This plate was also cemented to the pedestal and to the wall (see Figure 3a,c).

The set of TAPSs and CZ seismometer was connected to the “rotational” station MK-6, where the signals were sampled at 100 Hz. Such a sampling rate is sufficient for the strongest distant earthquakes and for regional seismic events, which include shocks in copper or coal mines. Hence, the records obtained with this station may be treated as similar to the effects of a low-pass filtering applied to much denser oscillations. Luckily, the elaborated data enabled us to reveal the hidden conformity between the signals from basically different sensors. Moreover, the possibility of a resonance occurring in each TAPSs, due to its three-feet suspension, cannot be excluded. Such a self-resonance (of other sensors) was described by Cranswick et al. [51] and the other authors cited therein. Its reported frequencies were well-above the Nyquist frequency of our TAPSs sensors, and we did not find a similar phenomenon in our research.

## 5. Results

**Figure 4:**
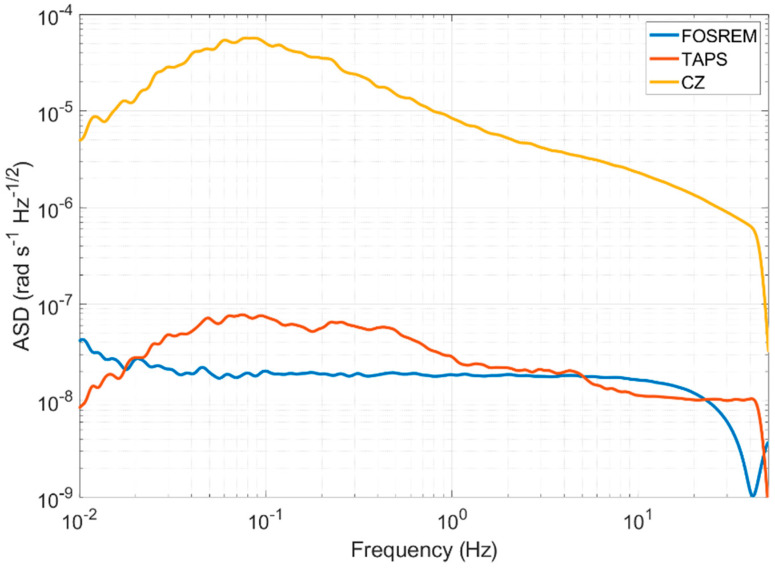
ASDs calculated for all sensors.

The rotation signals obtained during the mining works were recorded by all three types of the seismometers—the FOSREM, TAPS and CZ devices. An important factor that differed between these sensors was their resolution. In order to further compare the results from these devices, we first analyzed their self-noise, which is the output of the sensor when the sensor is at rest and no input motion is present. To estimate the instruments’ self-noise, we used recordings from several quiet nights in the Książ underground when no ground motion was detected. The calculated amplitude spectral density (ASD) characteristics for all three sensors are shown in Figure 4. It is worth noting that the ASDs were additionally filtered using a Konno-Ohmachi filter [52] with a smoothing coefficient equal to 40.

The FOSREM shows a typical flat self-noise spectrum in the range from 0.03 Hz to 20 Hz at the level of 20 nrad s^−1^ Hz^−1/2^, while the spectra of the TAPS and CZ sensor present a double-sloped curve with the maximum value at about 0.08 Hz–80 nrad s^−1^ Hz^−1/2^ and 0.5 mrad s^−1^ Hz^−1/2^, respectively. The overall level of ASD was comparable between FOSREM and TAPS, especially in the range from 1 Hz to 30 Hz. Even though, from 0.03 Hz up to 1 Hz, the self-noise spectrum of FOSREM was several times lower than the one for TAPS, both devices may be regarded as suitable for a weak rotation rate detection, with comparable resolutions. The self-noise characteristics of the CZ sensor clearly showed that this device is only useful for a strong motion recording.

Blast number 43 was chosen as an example of the rotational recordings obtained during the mining works. This was a multiple blast recorded at about 11:50 UTC on 23 April 2018. It was done from below at “Shaft 2” and obliquely oriented upwards. To perform the recordings analysis from devices with different sampling rates (656.168 Hz for FOSREM and 100 Hz for the TAPS and CZ sensor), first, all the signals were smoothed using a moving average with a 0.03-s window. Then, for the Pearson correlation coefficients calculation, the data from TAPS and CZ were up-sampled to the frequency of FOSREM. It is worth noting that the resampling was additionally preceded by applying an antialiasing filter to the signal using the Kaiser window method with the filter coefficients normalized to account for the processing gain of the window. The obtained signals were later time-shifted in order to find the best correlation coefficients between all the recordings. The obtained Pearson correlation coefficients are summarized in Table 1.

**Figure 5:**
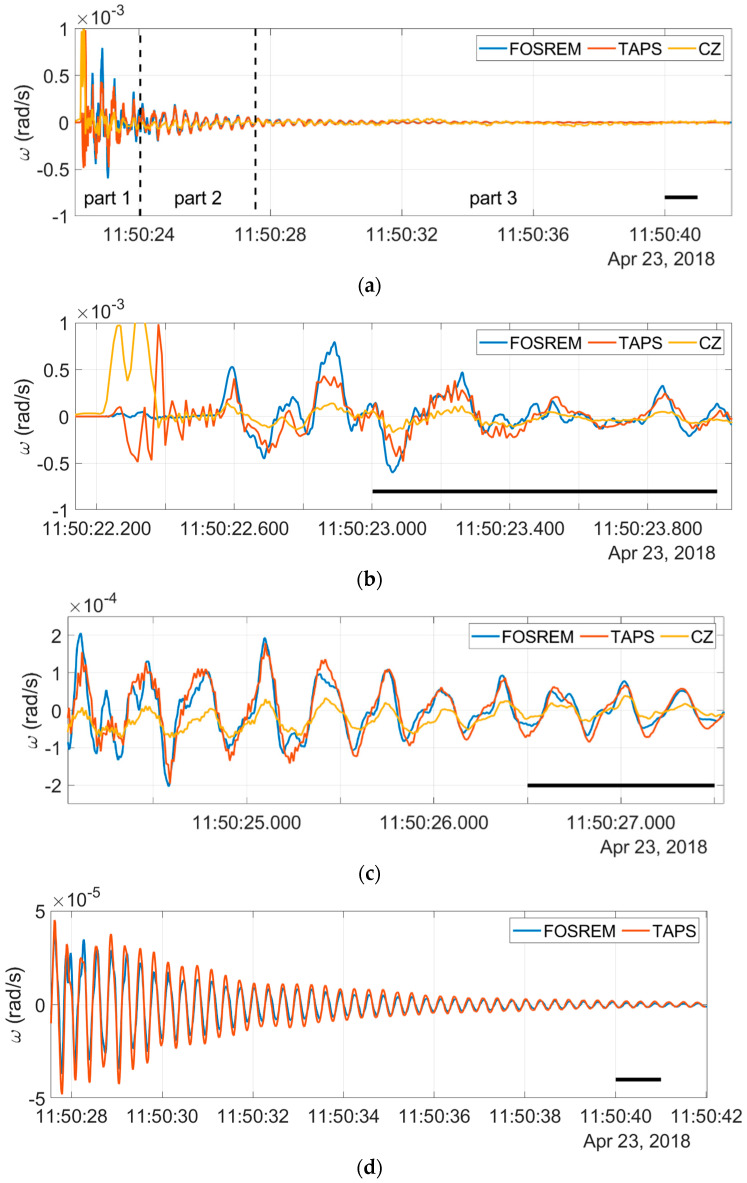
Rotation results from blast No. 43: (**a**) whole signal with dashed lines dividing three regions, (**b**) the first region with high amplitudes, (**c**) the second, intermediate, region and (**d**) the third region (without a CZ signal for better visibility). Thick black lines in the lower right corner denote a time of 1 s.

The signals presented in Figure 5 were only smoothed and time-shifted, without additional resampling, to better show the recorded characteristics. The whole registered signal (Figure 5a) was divided into three distinguishable regions: high-amplitude (>3 × 10^−4^ rad/s) and fast-changing beginning [Figure 5b], intermediate region (Figure 5c) and low-amplitude (<0.5 × 10^−4^ rad/s) and the slowly decaying final part in which all of the signals were well-matched except, for noisy data from the CZ sensor (Figure 5d).

In the first part of the aforementioned signals, the amplitude was fast-changing, which suggested a P-wave dominance in the elastic waves. The recordings presented in Figure 5b represent a similar waveform (except for the initial one of about 0.3 s), which is more evident for FOSREM and TAPS, while the signal from the CZ sensor is distorted but still follows the same low-frequency envelope. The second, intermediate, part represents the region where the signals’ amplitude starts to decrease and less high-frequency components are visible in the characteristics, as shown in Figure 5c. The best correlation between both the FOSREM and TAPS sensors is evident in the third part, where the amplitudes of both signals slowly fade away. Due to the high noise level of the CZ sensor in the third part, its data were omitted from presentation in Figure 5d. In this part, the registered signals were at a magnitude of 10^−6^ rad s^−1^, which was below the sensitivity of this instrument.

**Figure 6:**
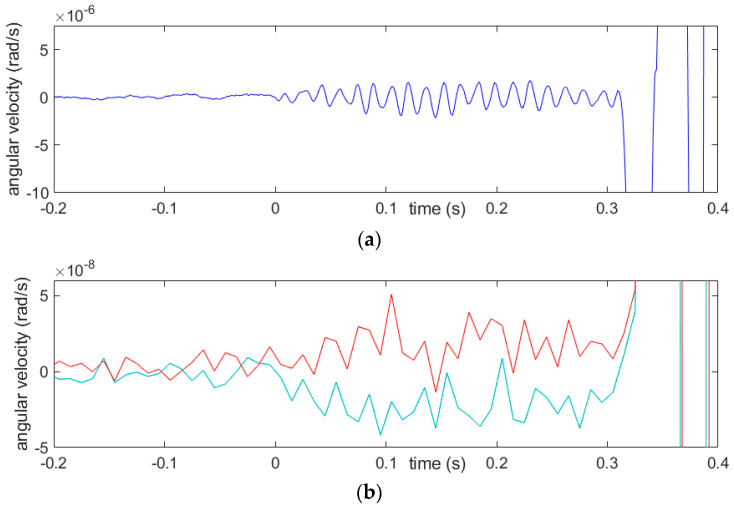
Low-amplitude initial part of the signal, as seen (**a**) in the recording from FOSREM and (**b**) in the symmetric strain rate (lower, turquoise line) and the rotation rate (upper, red line) calculated from the TAPSs. The solitary blast in corridor, No. 48, 26 April, was 73 g of the explosives.

In some registrations, mainly with FOSREM and additional Geosig stations (“linear” seismometers), the first characteristic low amplitude is visible after zooming in. It is located before the high-amplitude motions and suggests, at a glance, evidence of some technical action preceding the blast. The duration of this initial signal is, however, very short, about 0.3 s, and we consider such a section to be the first part of the actual registration of the blast, as is shown in Figure 6.

**Figure 7:**
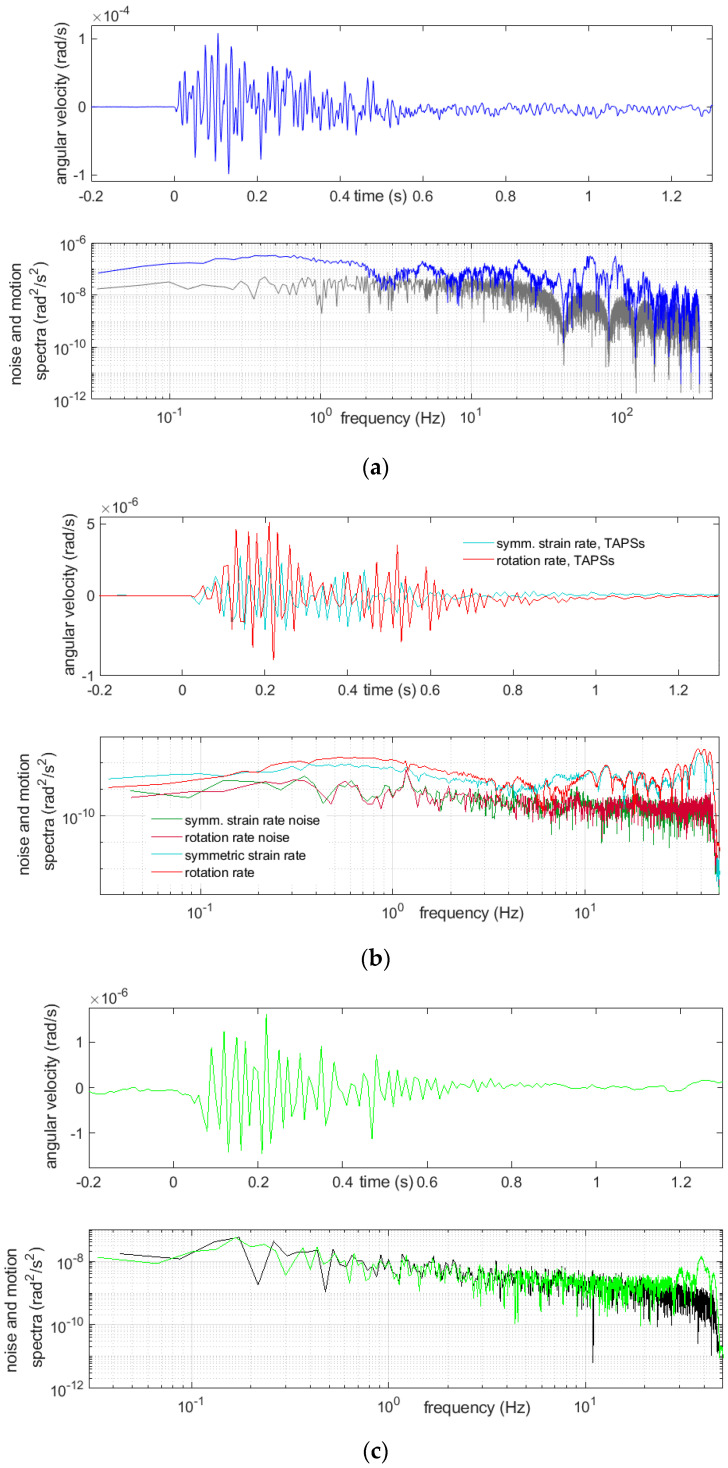
Recordings of the first burst on March 14th of 15 charges of 200 g, 15 time delays and total mass of the explosives 3000 g: (**a**) from FOSREM, the rotation rate and below its spectrum (blue), together with the rotational noise spectrum (gray), (**b**) from TAPSs, symmetric strain rate (turquoise) and rotation rate (red) and below the spectra of these motions, together with adequate noise spectra and (**c**) from the CZ sensor, rotation rate and below its noise (green), together with the rotational noise spectrum (black).

**Figure 8:**
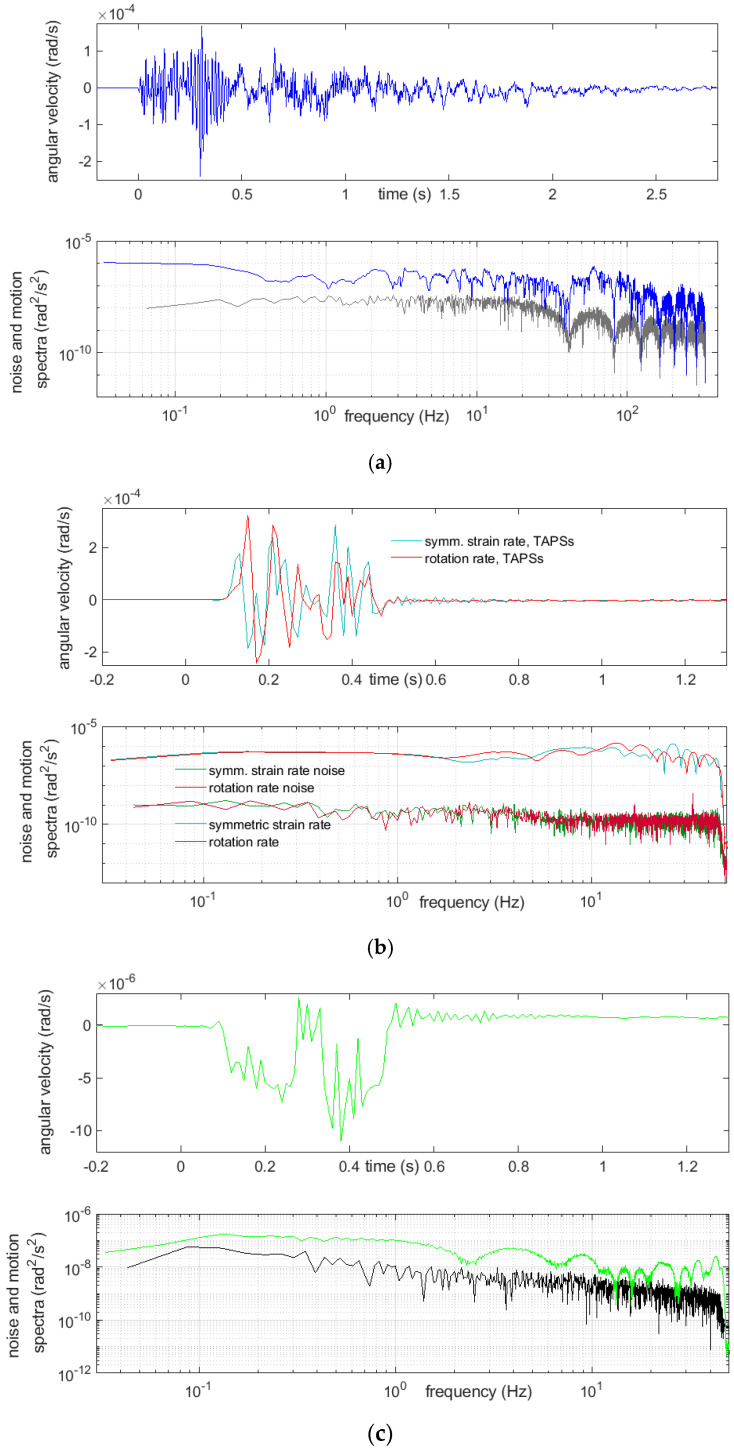
Recordings of a strong multiple blast, No. 39, on 19 April of 26 charges in 12 time delays and total mass of 9950 g: (**a**) from FOSREM, the rotation rate and below its spectrum (blue), together with the rotational noise spectrum (gray), (**b**) from TAPSs, the symmetric strain rate (turquoise) and rotation rate (red) and below the spectra of these motions, together with adequate noise spectra and (**c**) from the CZ sensor, the rotation rate and below its noise (green), together with the rotational noise spectrum (black).

**Figure 9:**
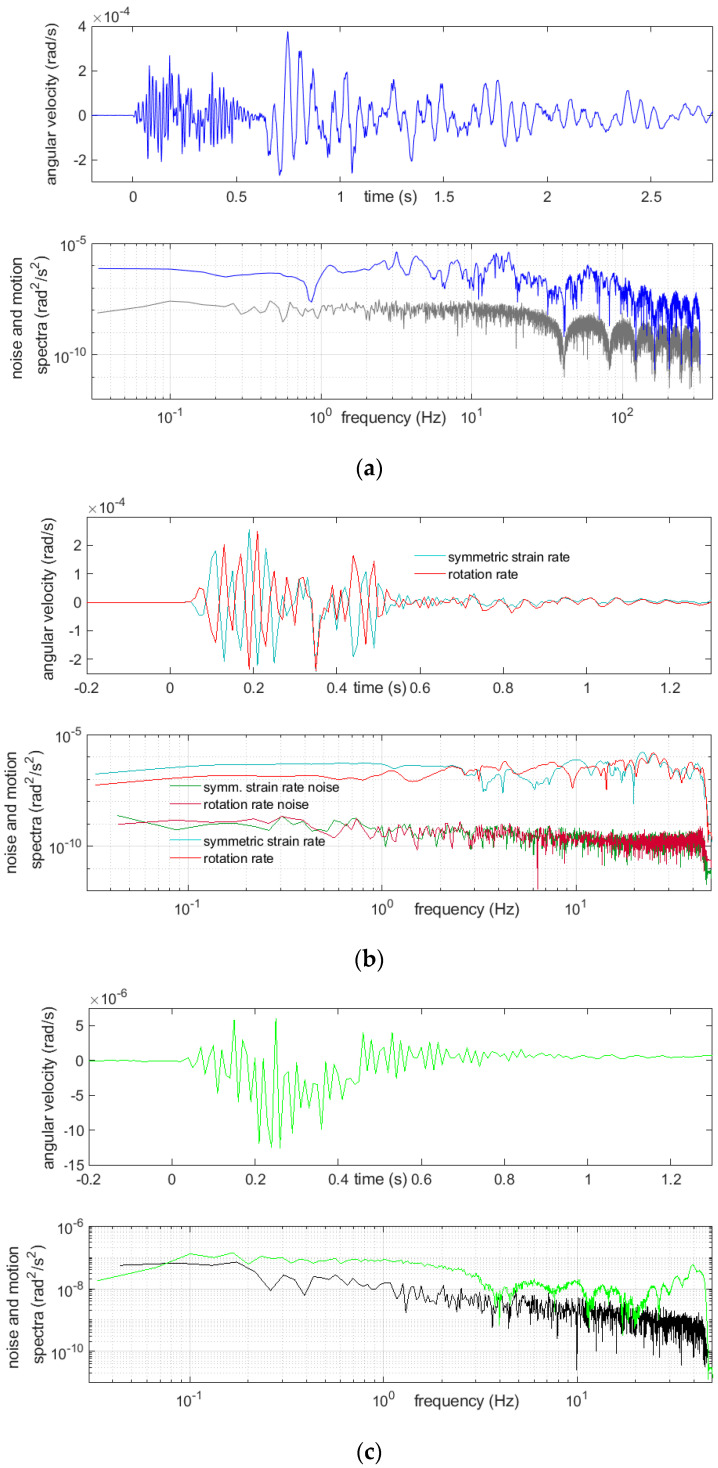
Recordings of the strongest multiple blast, No. 52, on 30 April of 30 charges in 15 time delays and a total mass of 12,750 g: (**a**) from FOSREM, the rotation rate and below its spectrum (blue), together with the rotational noise spectrum (gray), (**b**) from TAPSs, the symmetric strain rate (turquoise) and rotation rate (red) and below the spectra of these motions, together with adequate noise spectra and (**c**) from the CZ sensor, the rotation rate and below its noise (green), together with the rotational noise spectrum (black).

**Figure 10:**
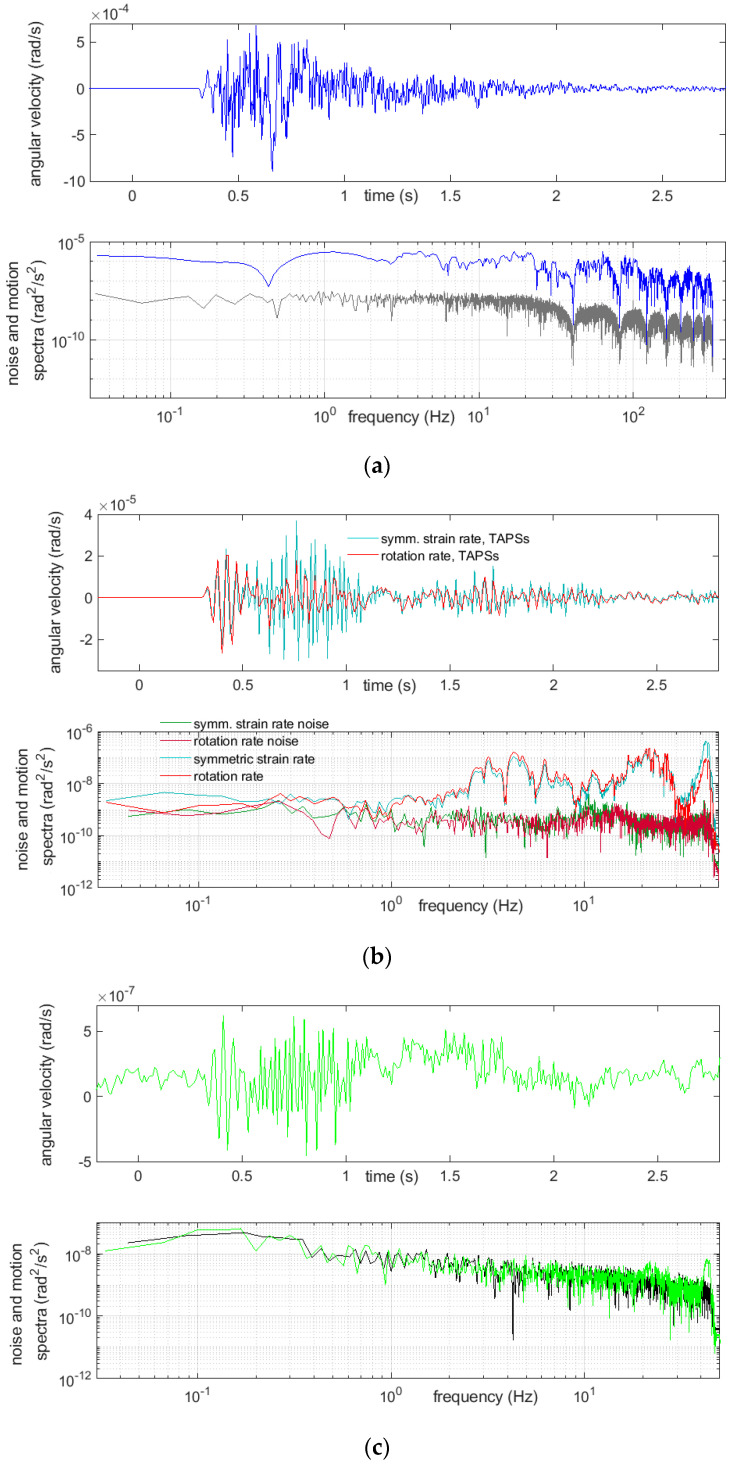
Recording of the solitary blast in the corridor, No. 48, on 26 April of 73 g of the explosive: (**a**) from FOSREM, the rotation rate and below its spectrum (blue), together with the rotational noise spectrum (gray), (**b**) from TAPSs, the symmetric strain rate (turquoise) and rotation rate (red) and below the spectra of these motions, together with adequate noise spectra and (**c**) from the CZ sensor, the rotation rate and below its noise (green), together with the rotational noise spectrum (black).

The timespan of the blast record in its first part did not exceed about 0.4 s–0.6 s, which was not much more than the greatest time delay of the elemental explosion (0.375 s). This was to be found more precisely in the records from FOSREM—see Figure 5 and Figure 7, Figure 8, Figure 9 and Figure 10. This first part of the record was followed with a lower-frequency part, which passed into the coda, sometimes very slowly decaying.

The last liquid-filled CZ sensor has too low of a sensitivity in everyday practice. The strongest mining shocks in the Lower Silesia Copper Basin, having a local magnitude of 3 and higher, are recorded in a readable form; smaller shocks make recordings excessively blurred with noise. In our study, a rotation was also clearly seen in the recordings from this device, but here, its amplitudes were about 1/15 of those obtained from the other rotational sensors. After recording the shock itself, a very long coda followed, resembling those present on the FOSREM recordings but struck by noise.

Examples of registration of two very strong multiple blasts and the above-mentioned boulder-crushing blast are shown below (Figure 7, Figure 8, Figure 9 and Figure 10). The main characteristics of the bursts that are shown or mentioned in this article are summarized in Table 2.

The spectra, shown in Figure 7, Figure 8, Figure 9 and Figure 10, were calculated by taking a 30-s time interval starting at a whole number 24 s before the event-induced waves arrived at the receiver. Therefore, each of three intervals embraced slightly more than 6 s of the shock and did not cover the full coda—see Discussion and Conclusions.

A relatively long quiet time period before the event was taken into the calculations for improving the spectra accuracy. Beside the rotational motion spectra, the spectra of the rotational noise were also measured; these were usually based on the first 23 s of the same chosen interval. A considerable variability of the spectra was seen from these examples. The reactions of the CZ sensor were dominated with low frequencies and, also, high ones extending 45 Hz, which were, of course, not exactly represented in the data, because the Nyquist frequency was 50 Hz for this sensor. Except for the highest frequencies allowed by the sampling, the recordings from this sensor appeared to be at the noise level. Often, recordings from this sensor showed low-frequency (below 2 Hz) and low-amplitude irregular undulations, while simultaneous recordings from the other sensors did not; however, a comparison of the spectra of the noise and signal from this device showed that this effect cannot be treated as reliable. The signal spectra from the other sensors showed a high content of relatively low frequency rotational vibrations, around 20 Hz and below, which was to be expected.

The main parameters of the particular blasts that are discussed in this article are summarized in Table 2. “S” means that the blast was coming from the surface and was directed downwards (arrow pointing down), while “B” means that the blast was placed underground below the surface and was directed upwards (arrow pointing up).

**Figure 11:**
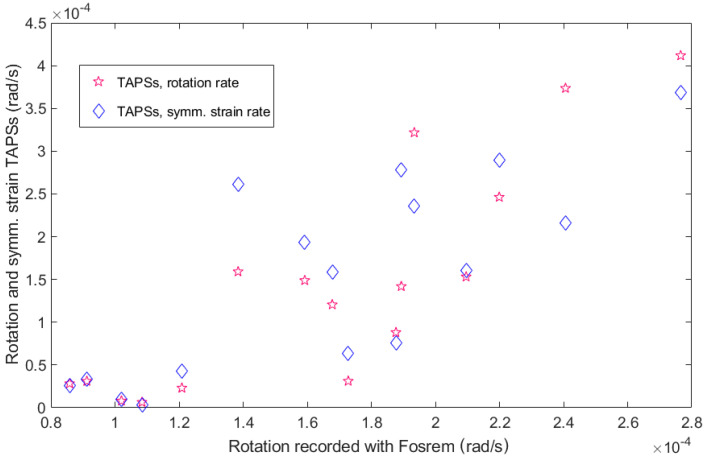
Maximum velocities of the rotation and symmetric strain motions in the rock mass, obtained from two sets of TAPS, were plotted against the maximum velocities of the rotational motions recorded with FOSREM due to the same 16 multiple blasts directed vertically from above.

In the blasts’ seismic effects, as we deduced from the seismograms from various devices, for the 16 blasts directed vertically from above, there was a visible correlation between peak ground rotational motions found with FOSREM and TAPS (both rotation and symmetric strains)—see Figure 11. For a better comparison of the maximal velocities recorded by different types of devices, a Pearson correlation coefficient was calculated for each pair of sensors, and the results are summarized in the upper part (above the diagonal) of Table 3.

**Figure 12:**
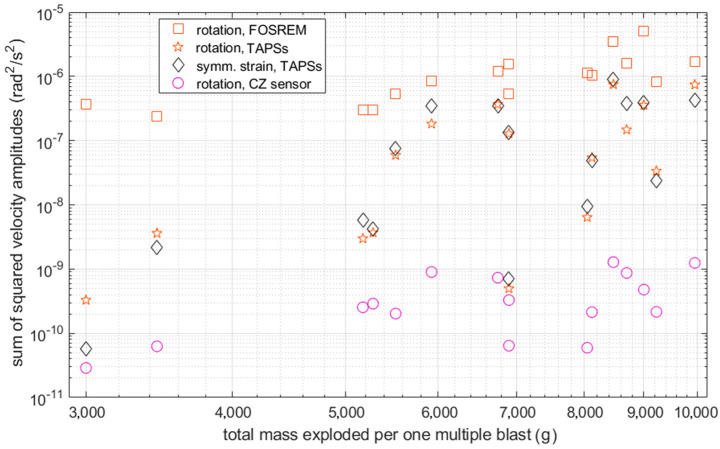
Sums of the squared velocity amplitudes of: rotations recorded with FOSREM and with the set of TAPSs, symmetric strain from the latter and rotation recorded with the CZ sensor with liquid. All are shown in (rad/s)^2^ and plotted against the total masses in grams per each of the studied 16 multiple blasts that were directed vertically down. Signals from all the sensors except FOSREM were multiplied by suitable coefficients, as shown in the legend, in order to make their relative position visible.

There is also a certain dependence of the sum of the squared amplitudes on the explosive mass fired in a particular multiple shot—see Figure 12 and Table 3, where, in the lower part (below the diagonal), the summarized values of the Pearson correlation coefficients for each pair of sensors are shown. The sum of the squared amplitudes had to be taken into account, because the radiated seismic energy depended on it (while the spatial and temporal structures of the source modified the relation, and the received energy also depended on the propagation path). For this comparison, a time period of 3 s was taken for each event.

It was evident that, even for the TAPS rotation and strain comparison, the Pearson correlation coefficient was not very close to 1, with a maximum value of about 0.90. Nevertheless, the presented values were calculated for datasets of only 16 samples, so obtaining a correlation above 0.70 already suggests a good agreement between the results from different sensors. The relatively lower correlation values obtained for the sums of the squared velocities between FOSREM and the other sensors may be attributed to a different sampling frequency, which was over 6.5 times higher than for TAPS and the CZ sensor. On the other hand, the slightly lower correlation values calculated for the maximum velocities between the CZ sensor and the others were most probably due to the lower sensitivity of this device, which caused it to be useful only for strong high-amplitude ground motions.

On the other side, we found only a weak correlation between the signal extremes and the total explosive mass fired in one multiple blast or even the mass that was fired at the same moment. The inclusion of multiple blasts applied from below or one small solitary blast in the underground corridor blurred each scheme’s relationships and was therefore omitted from this paper.

## 6. Discussion and Conclusions

By examining the rotational recordings of the studied blasts, it was impossible to isolate the effects of the consecutive elemental blasts, and only in some cases was it possible to perceive the approximate arrival of the “phases”; parts of the wave field were dominated, e.g., with S-waves. There were similar concerns for the arrival of boundary waves (created by an interference of the waves reflected at the corridor floors and ceilings, with those reflected from the soil surface). These issues are inevitable in such short-distance observations.

As could be seen in the selected examples, the blast-induced angular motions were very variable. The signal shape varied between the cases, and this was to be expected: the weight of the explosive, proportion and sequence of separately fired parts differed from one blast to another. Additionally, the rock mass reaction was, to some extent, unpredictable. Nonetheless, the shapes of the obtained recordings and their parameters may even seem to be chaotically diversified. Hence, the main question we were trying to answer was: what is the cause of so much variability in the results and the lack of order, despite knowing the basic parameters of the sources.

At first, it was important to notice that the blasts generated linear oscillations that were stronger than those radiated by more distant (copper or coal mining) events in the area, and the angular motion velocities were even one order of magnitude higher than those caused by the latter. The lack of a strong relation between the extremes of the signals obtained from various instruments was initially disappointing. All this pointed to the high variability of the seismic wave field. We suppose that its parameters differed slightly even at inter-sensor distances. We attributed this to the complex character of the blasts, then to the interference of many reflections from the oblique and uneven soil surface with those arriving directly. At such a short distance, we excluded the contribution of the reflections and bending at that depth. In summary, waves of slightly different history reached each of the sensors. Nevertheless, the compliance of the rotation and symmetric strain obtained from the set of TAPSs showed that nothing extraordinary happened to the sensors. Additionally, each elemental blast was, in fact, slightly different and unique, as was their interplaying in the source; the induced seismic wave fields differed as well.

The relationship between the sum of the explosive mass used for one compound (multiple) blast and the sum of the squared signal amplitude (Figure 12) was a measure of how the energy added to the system affected the energy received (at a given rotation) in the form of oscillations. This relationship was influenced by the source area parameters (which varied) and the details of each compound blast. Nevertheless, the overall correlation of all the devices was relatively high (taking into account only 16 samples), especially for the ones sampled with the same frequencies (TAPS and CZ). The lower correlation values obtained between FOSREM and the other sensors may be attributed to the higher sampling frequency of this instrument. Another important factor of the blasts located in close proximity to the analyzed sensors was the maximum angular velocity of the registered signals. Again, the overall correlation between all the devices could be considered relatively high, although significantly lower for the CZ sensor compared to the others due to the much lower sensitivity of this device.

The codas of the obtained signals were asymmetric. This asymmetry and the length of this part of the signal, reaching several tens of seconds, were both caused by the asymmetric impulse, which manifested mainly in the coda and came from the nearby field from the rock mass motion around the source.

The fact that the inclusion of multiple blasts applied from below or a single blast in the underground corridor broke the relationship patterns obtained for the 16 blasts applied from above indicates some yet undisclosed features of the induced seismic fields.

It is difficult to assess the uncertainty of the results. Of course, the sensors differed slightly, but in our opinion, there was no indication of this fact being the cause of the results’ variability. On the other hand, the evident spatial variability of the induced seismic wave field masked the inevitable non-identities of the sensors. This was related to the TAPSs and their inner elements. The limitations of the registration as a whole include the nonuniform characteristics and too-narrow frequency registration scale in the case of the TAPSs and CZ sensor.

In conclusion, we registered and analyzed the rotational movement signals from multiple blasts during mining works in the underground of the Książ Castle hill. The data obtained from three different types of rotational seismometers were compared and analyzed in terms of their self-noise, spectral characteristics and rotational velocities. Based on the results presented for FOSREM and TAPS, we can conclude that they are in a good agreement with each other, and both of these sensors can be used for the registration of weak rotational movements, even though TAPS is a much older device. The registrations from the CZ sensor had serious drawbacks—a low sensitivity and high level of a low-frequency noise. Once corrected, this device may be used for strong motion observations from a short distance.

While the TAPS and CZ sensor may be regarded as closed projects, the FOSREM family of devices is in a constant state of development with the aim to provide a robust mobile platform capable of detecting the rotational motions from natural and artificial sources in the most crucial and vulnerable infrastructures–e.g., mines, power and processing plants, bridges or even skyscrapers. The main direction of the further development of this type of device is to improve its sensitivity and lower the self-noise characteristics. In addition, in its current state, the FOSREMs are designed to measure only one component (vertical) of the rotational ground motions. An obvious expansion of this idea would be to add two more perpendicular fiber loops to provide a solid and reliable platform for detecting angular oscillations in all three dimensions.

## Figures and Tables

**Table 1:** Pearson correlation coefficients between all the sensors.

	FOSREM	TAPS	CZ
**FOSREM**	-	0.96 ^3^	0.70 ^2^
**TAPS**	0.64 ^1^	-	0.75 ^2^

^1^ Calculated for part 1. ^2^ Calculated for part 2. ^3^ Calculated for part 3.

**Table 2:** Main parameters of the chosen blasts that are mentioned in this article.

No.	Shaft/Orientation	Date	Time UTC	Quantity of Detonators	Quantity of Time Delays	Mass of Charges (g)	Total Mass of Charges (g)
1	3/S ↓	14 March 2018	10:09	15	15	200	3000
2	3/B ↑	14 March 2018	12:03	20	15	240	4800
39	2/S ↓	19 April 2018	10:01	26	12	375; 400	9950
43	2/B ↑	23 April 2018	11:50	14	12	375	5250
48	2/B ↑	26 April 2018	06:45	1	1	73	73
52	3/B ↑	30 April 2018	10:01	30	15	400; 450	12,750

**Table 3:** Pearson correlation coefficients of the maximal angular velocities and sums of the squares of the angular velocities between all pairs of the sensor datasets.

	FOSREM	TAPS Rotation	TAPS Strain	CZ
**FOSREM**	-	0.86 ^1^	0.80 ^1^	0.60 ^1^
**TAPS rotation**	0.65 ^2^	-	0.86 ^1^	0.71 ^1^
**TAPS strain**	0.72 ^2^	0.90 ^2^	-	0.76 ^1^
**CZ**	0.49 ^2^	0.89 ^2^	0.90 ^2^	-

^1^ Calculated for the maximum angular velocities (see Figure 11). ^2^ Calculated for the sums of the squared angular velocities (see Figure 12).

## Data Availability

The datasets generated and analyzed during the presented study are available from the corresponding authors upon reasonable request.
